# Experimental Designs to Study the Aggregation and Colonization of Biofilms by Video Microscopy With Statistical Confidence

**DOI:** 10.3389/fmicb.2021.785182

**Published:** 2022-01-13

**Authors:** Brian A. Pettygrove, Heidi J. Smith, Kyler B. Pallister, Jovanka M. Voyich, Philip S. Stewart, Albert E. Parker

**Affiliations:** ^1^Center for Biofilm Engineering, Montana State University, Bozeman, MT, United States; ^2^Department of Microbiology and Cell Biology, Montana State University, Bozeman, MT, United States; ^3^Department of Chemical and Biological Engineering, Montana State University, Bozeman, MT, United States; ^4^Department of Mathematical Sciences, Montana State University, Bozeman, MT, United States

**Keywords:** microscopy, biofilm, image analysis, statistical confidence, repeatability, experimental design

## Abstract

The goal of this study was to quantify the variability of confocal laser scanning microscopy (CLSM) time-lapse images of early colonizing biofilms to aid in the design of future imaging experiments. To accomplish this a large imaging dataset consisting of 16 independent CLSM microscopy experiments was leveraged. These experiments were designed to study interactions between human neutrophils and single cells or aggregates of *Staphylococcus aureus* (*S. aureus*) during the initial stages of biofilm formation. Results suggest that in untreated control experiments, variability differed substantially between growth phases (i.e., lag or exponential). When studying the effect of an antimicrobial treatment (in this case, neutrophil challenge), regardless of the inoculation level or of growth phase, variability changed as a frown-shaped function of treatment efficacy (i.e., the reduction in biofilm surface coverage). These findings were used to predict the best experimental designs for future imaging studies of early biofilms by considering differing (i) numbers of independent experiments; (ii) numbers of fields of view (FOV) per experiment; and (iii) frame capture rates per hour. A spreadsheet capable of assessing any user-specified design is included that requires the expected mean log reduction and variance components from user-generated experimental results. The methodology outlined in this study can assist researchers in designing their CLSM studies of antimicrobial treatments with a high level of statistical confidence.

## Introduction

Biofilms consist of matrix embedded aggregates of microorganisms, are widespread in natural and engineered environments, and are physiologically distinct from free-living communities. Biofilm development begins with the loose association of microbes, most often on a surface, followed by a more robust cellular adhesion and aggregation (Hall-Stoodley et al., 2004). The process of biofilm development is an active area of research that has been advanced by modeling, imaging, and “omic” based approaches (de Beer et al., 1994; Sauer et al., 2002; Rani et al., 2005; Connolly et al., 2015). One of the most important developments in the study of biofilm formation, structure, and dynamics has been the application of optical imaging, specifically confocal laser scanning microscopy (CLSM). CLSM has the ability to provide 3D information of hydrated intact biofilms non-invasively and in real-time (Stewart et al., 2009; Davison et al., 2010; Franklin et al., 2015). CLSM studies have provided valuable insight into biofilms including localization of gene expression (McLoon et al., 2011; Hung et al., 2013), analysis of extracellular material (Allesen-Holm et al., 2006; Baird et al., 2012; Gloag et al., 2013), community organization (Brileya et al., 2014), flow patterns (Martínez-García et al., 2018), the diffusivity of solutes (Stewart et al., 2009), and the spatio-temporal patterns of biocide action (Bridier et al., 2011), making it a powerful tool for any biofilm researcher. However, when studying developing biofilms, the potential for increased variability within early stage biofilms in combination with phototoxicity increases the need for the development of methodological approaches that carefully consider all steps of an imaging experiment including experimental design, data collection, and the reporting of results (Lee and Kitaoka, 2018).

It is standard practice in biological research to establish the repeatability and reproducibility of a particular technique or method and this trend has extended into the biofilm field. There is increasing recognition of the importance of standardized methods in biofilm research to ensure reproducibility across labs (Malone et al., 2017). Efforts have been made to develop standard methods for several biofilm assays (Gomes et al., 2018) such as the drip-flow reactor (Goeres et al., 2009, 2020), single-tube method (Goeres et al., 2019), the CDC biofilm reactor (Goeres et al., 2005), and the 96 well MBEC assay (Parker et al., 2014). However, studies recommending standard practices for imaging-based biofilm experiments have not been similarly reported. A comprehensive review by Magana et al. (2018) found that biofilm methodology was under-represented as a percentage of all published papers in the field with only 4.9% of papers focusing on assay or method development. Conversely, for the field of genome editing, they found 23.4% of papers to be related to methodology. We have searched the biofilm literature for papers on the topic of biofilm imaging methods and observed that the majority of biofilm imaging methods papers are dedicated novel image analysis techniques and packages that assist researchers in quantifying biofilm characteristics such as volume, structure, and surface coverage (Heydorn et al., 2000; Yang et al., 2000; Daims et al., 2006; Mueller et al., 2006; Yerly et al., 2007; Milferstedt et al., 2009; Renslow et al., 2011; Almstrand et al., 2013; Sommerfeld Ross et al., 2014; Tolker-Nielsen and Sternberg, 2014; Larimer et al., 2016; Vyas et al., 2016; Baudin et al., 2017; Luo et al., 2018, 2019; Parker et al., 2018b, 2020). While these studies are essential to further the field of biofilm research by improving the quality and accuracy of data acquired through imaging, we are aware of few papers that include any recommendations for the design of biofilm imaging experiments to assure that data are representative, repeatable across experiments, and reproducible across labs (Korber et al., 1993; Lawrence and Neu, 1999; Heydorn et al., 2000; Daims and Wagner, 2011; Menzel et al., 2016). To answer questions of interest from imaging data with a high level of statistical confidence, a quantitative assessment of variability in biofilm imaging data that specifies the requisite number of experiments and fields of view (FOV) is necessary but currently is lacking. To achieve statistical confidence, there is often an inclination to collect as many FOVs as possible. This approach has the potential to negatively impact data quality in several ways. Excessive imaging of a sample can result in photosensitivity of fluorophores and decrease data quality, although next generation imaging technologies such as light-sheet microscopy are beginning to mitigate these concerns somewhat (Power and Huisken, 2017; Qin et al., 2020). Increasing the number of FOVs being imaged can decrease the temporal resolution for time lapse microscopy, which can be a large detriment when studying rapid processes. Furthermore, advancements in imaging technology which have allowed researchers to generate massive amounts of data by imaging many FOVs over high temporal resolutions require a significant amount of time and computational resources to process and analyze collected images. Thus, identifying the correct balance between sample number and statistical confidence can make imaging-based experiments more efficient while maintaining high quality data and providing confidence in the downstream interpretation.

This paper provides guidance on designing experiments and interpreting resulting data from time-lapse visualization studies with an emphasis on early colonization biofilms. Recommendations are given for the number of experiments, FOVs and frames per hour that should be collected for imaging studies. Data were mined from 16 independent CLSM microscopy experiments that were designed to study interactions between human neutrophils (polymorphonuclear leukocytes or PMNs) and single cells or aggregates of *S. aureus* during the initial stages of biofilm formation. These data, including biological replicates (i.e., independent experiments) and varying inoculum concentrations, were then analyzed to identify the variability associated with the different levels of replication. We use the results to suggest future microscopy experimental designs. Based on the variability identified with each level of replication, results presented herein allowed us to recommend how often to take CLSM images (i.e., the temporal resolution of the sequence of images), how many FOVs to include, and how many replicate experiments to perform when studying initial attachment of biofilms using surface coverage as a quantitative response. While the presented analysis and results are specific to the data set described above, any pilot data can readily be analyzed using the approach that we present to inform experimental designs. We include our experimental design assessment tool as an Excel spreadsheet in the Supplementary Materials.

## Methods

### Bacteria and PMN Preparation

*Staphylococcus aureus* strain AH2547 (HG001 + pCM29, courtesy of Alex Horswill), a known biofilm-forming strain (Pabst et al., 2016) with constitutive expression of green fluorescent protein (GFP), was grown overnight in tryptic soy broth supplemented with 10 μg/ml chloramphenicol for maintenance of the GFP carrying pCM29 plasmid. Overnight cultures were centrifuged for 5 min at 4,000 rpm, rinsed, resuspended in phosphate buffered saline (PBS), and serially diluted. Cells were attached to a 4-chambered glass bottom petri dish (Cellvis, CA, USA) to facilitate live imaging. To attach bacteria, 10 μL of diluted bacterial suspension were added to the surface. After 30 min of incubation at 37°C, unattached bacteria were gently rinsed from the surface with PBS. Each chamber of the petri dish was filled with 1 ml of 10% fresh human serum in Hank's Balanced Salt Solution (HBSS) with Ca^2+^ and Mg^2+^ and incubated at 37°C for 30 min. PMNs were isolated from multiple heparinized venous blood obtained from healthy donors following a standard IRB-approved protocol as described previously (Voyich et al., 2005, 2009). All donors provided written informed consent to participate in the study. PMNs were isolated under endotoxin-free conditions (<25 pg ml^−1^) and purity (<1% PBMC contamination) and viability (<2% propidium iodide positivity) of PMN preparations were assessed by flow cytometry as previously described (Voyich et al., 2005, 2009). PMNs were kept on ice until stained with LysoBrite Red (AAT Bioquest, CA, USA) according to the manufacturers instructions. Stained PMNs were added to the appropriate wells and interactions between *S. aureus* and PMNs were imaged for 4 h. Some experiments utilized PMNs from the same donor, although PMNs from the same donor were always collected and prepared on different days.

For the antibiotic treatment assay, *S. aureus* was attached to the petri dish surface as described above, grown for 3 h in 10% human serum in HBSS, then challenged with 10 μg/mL gentamicin and imaged for 4 h.

### Microscopy

A Leica SP5 inverted confocal laser scanning microscope was utilized for all imaging. GFP-tagged bacteria and PMNs were excited with the 488 nm and 561 nm laser lines, respectively. A LiveCell (Pathology Devices, CA, USA) environmental chamber system was utilized to maintain 5% CO_2_, 20% O_2_, 50% humidity, and 37°C for sample incubation during imaging. Image stacks 12–20 μm in size with 1-μm z-slices were taken sequentially at 2–3 min intervals over a 4-h time course using a Leica 20x/0.7 NA dry objective lens. At least two fields of view from each chamber of the dish were generally imaged per experiment. FOVs with large numbers of bacterial objects were selected for imaging to visualize the highest number of *S. aureus*—PMN interactions.

### Image Analysis

Images were analyzed as maximum projections of z-stacks at each time point. MetaMorph v 7.8.13 (Molecular Devices) image analysis software was used to measure change in bacteria biomass by quantifying the thresholded area of bacterial green fluorescence in each collected image. Log reductions (LR) in GFP area for bacteria treated with PMNs were calculated for each time point using the formula

$$\begin{array}{l} LR=\log_{10}\left(Control\right)-\log_{10}\left(Treated\right) \end{array}$$

where the control area was an average area of the two fields of view from the control well for a given experiment and the treated area was of a single field of view of interest.

### Repeatability Analysis

A linear mixed effects model (LMM) was fit to each hour of data [either log_10_(GFP) area of the controls or LRs] separately, with FOV nested in Experiment as random effects and time as a covariate. This LMM estimated the components of variance due to experiment (Var_exp_), FOV (Var_FOV_) and time (Var_time_). To pool the time periods in each approximate phase of growth, an LMM was fit to data from each phase with fixed effects for log(Inoculum), time period and the two-way interaction, and covariates for time as well as all 2 and 3 way interactions with the fixed effects. The serial correlation over time was modeled with an autoregressive process of order 1 [AR(1)] by fitting the LMM and then inflating the variance for the error term using the approach described in section 15.2 of Ramsey and Schafer (2013). This variance inflation factor is $c=\frac{1\;+\;r}{1\;-\;r}$ that depends on the first *serial correlation coefficient* $r=\frac{\overset{n}{\underset{t=2}{\sum }}res_{t}\times res_{t-1}}{\overset{n}{\underset{t=1}{\sum }}res_{t}\times res_{t}}$ where *res* is the set of LMM residuals. Quadratic trend was fit to the variances as a function of the LR as described by Parker et al. (2018b). All LMMs were fit in R v4.0.1 package lme4 (Bates et al., 2015).

### Experimental Designs

The main question of interest is how to use the repeatability results from the LMM to answer questions about microscopy experimental design. To answer this question, we focus on the precision of the mean response [either log_10_(GFP area) or LR] given by the standard error of the mean (SEM) and a one-sided 95% CI of the mean. The equation for the SEM from an experimental design with *n*_*exp*_ experiments, *n*_*FOV*_ FOVs per experiment and *n*_*time*_ per FOV is

(1) $$\begin{array}{r} SEM=\sqrt{\frac{Var_{exp}}{n_{exp}}+\frac{Var_{FOV}}{n_{exp}n_{FOV}}+\frac{Var_{time}\times c}{n_{exp}n_{FOV}n_{time}}} \end{array}$$

Equation (1) is the standard formula for the SEM when the data are balanced and there is no serial correlation over time (i.e., when *r* = 0 and the variance inflation factor is *c* = 1). The variance inflation factor is *c* > 1 when the data over time are correlated in which case the SEM is inflated to account for that correlation. In other words, as the variance inflation factor *c* increases due to higher correlation over time, the SEM also increases through the third term in equation (1). We estimated the variance components Var_exp_, Var_FOV_, and Var_time_ in equation (1) from our data using the LMM described above. Our data can be used to estimate the SEM when the images are collected at a single time point (i.e., there is no video) by setting n_time_ = 1 in equation (1). If one wants to use their own data for single time point image data, then one would fit an LMM with a single random effect for experiment, retrieve variance components for Var_exp_ and Var_FOV_, and plug in values for n_exp_ and n_FOV_ and set Var_time_ = 0 in equation (1).

For any growth phase given in Table 1, we can fill in the values in equation (1) to get the SEM for the mean log(GFP area) for the untreated control biofilms. For example, for an experiment that is run to the early exponential phase (see row 2 in Table 1), the SEM is


$$\begin{array}{l} SEM\\ =\sqrt{\frac{0.583\times 0.3186^{2}}{n_{exp}}+\frac{0.403\times 0.3186^{2}}{n_{exp}n_{FOV}}+\frac{0.0137\times 0.3186^{2}\times 15}{n_{exp}n_{FOV}n_{time}}}. \end{array}$$

**Table 1:** Repeatability standard deviation for each phase of biofilm growth and the proportion (Prop.) of variance attributable to each of experiment, field of view, and time.

**Phase**	**Time Period (h)**	**Prop. Exp**	**Prop. FOV**	**Prop. Time**	**Repeat. SD**	**First serial correlation**	**Inflation factor**
Lag	[0, 2]	0.3207	0.6707	0.0086	0.4867	0.8313	10.9
Early	[2, 4]	0.5831	0.4033	0.0137	0.3186	0.8751	15.0
Late	[4, 8]	0.4540	0.5151	0.0309	0.2148	0.9129	22.0

*The closer that the serial correlation r is to 1, the more dependent the data are over time. The variance inflation factor indicates how much the serial correlation over time increases the SEM via equation (1)*.

To fill out the previous formula:

- If an image is collected every 3 min, then 20 images are collected in each FOV over an hour-long experiment. So the number of images inputted into the formula is *n*_*time*_ = 20.
- If 3 FOVs are collected per experiment, then *n*_*FOV*_ = 3.
- If there are 2 independent experiments then *n*_*exp*_ = 2.

Given this setup, then the SEM in this example is

$$\begin{array}{l} SEM=\sqrt{\frac{0.583\times 0.3186^{2}}{2}+\frac{0.403\times 0.3186^{2}}{2\times 3}+\frac{0.0137\times 0.3186^{2}\times 15}{2\times 3\times 20}}\\ \;=0.191. \end{array}$$

In this example the correlation over time inflates the time component of the SEM by the variance inflation factor *c* = 15 (Table 1). For these control data collected from the early exponential phase the variance component due to time is small (≤ 1.4%, see Table 1), as is commonly the case, so the SEM remains about the same size whether or not the correlation due to time is accounted for [i.e., whether, in equation (1), *c* > 1 as in Table 1 or *c* = 1].

The lower limit for an upper one-sided 95% confidence interval (CI) for either the mean control response or the mean LR is calculated by

$$\begin{array}{l} Mean-t\;\times \;SEM. \end{array}$$

The multiplier *t* is the 95th percentile from a *t* distribution with *n*_*exp*_ − 1 degrees of freedom. In our results, we consider *n*_*exp*_ = 1, 2, 3, and 6 and provide the margin of error, MOE = *t* × *SEM*, for a one-sided 95% confidence interval for each of these scenarios coupled with specifications for *n*_*time*_ = 10 or 40, and *n*_*FOV*_ = 1, 2, and 6. When *n*_*exp*_ = 1, we use a *t* distribution with 0.5 degrees of freedom to give an approximate MOE for a 1-sided 95% CI for the mean. For all SEM and MOE calculations for the mean LR, the inflation factor *c* = 12 was used. This value is the maximum possible value observed for our LR data (see Supplementary Material).

The SEM in equation (1) is closely related to the repeatability SD that quantifies the variability of the data across time points, FOV and experiments, by

(2) $$\begin{array}{r} \text{Repeatability SD}=\sqrt{Var_{exp}+Var_{FOV}+Var_{time}\times c} \end{array}$$

Equation (2) shows that when the data over time are correlated, then the repeatability is inflated to account for that correlation. In other words, as the variance inflation factor *c* increases due to higher correlation over time, the repeatability SD also increases through the third term in equation (2).

## Results

### Experimental Setup and Data Acquisition

**Figure 1:**
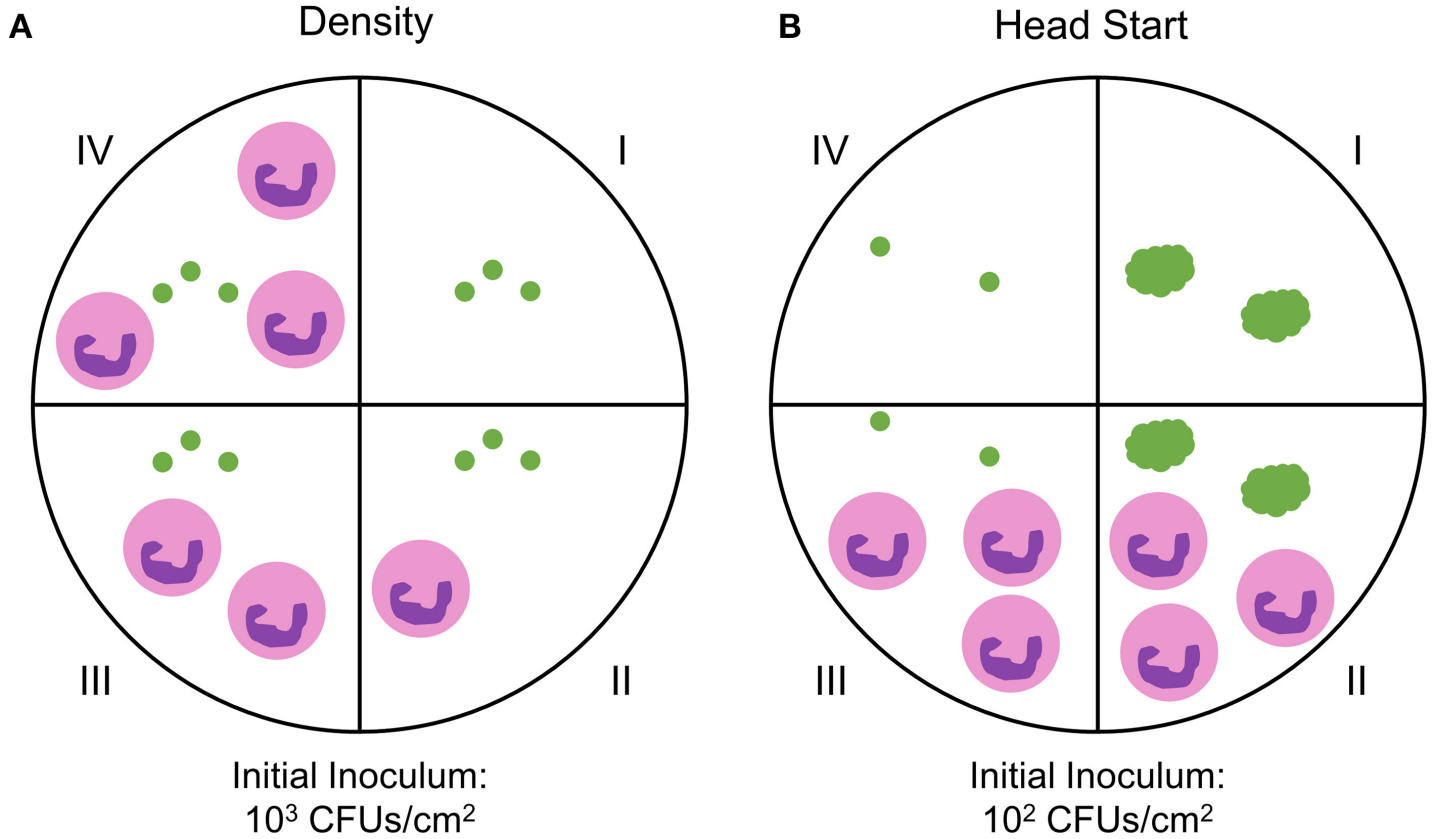
Experimental setup for probing PMN-*S. aureus* interactions. Two FOVs were imaged in each well unless otherwise noted. **(A)** *S. aureus* cells were attached to the dish surface at an initial inoculum of ~10^3^ CFUs/cm^2^. A single well-contained *S. aureus* only (green dots, well I) while all other wells received *S. aureus* and PMNs (pink circles, wells II - IV). *S. aureus*—PMN interactions were then imaged for 4 h. *N* = 10 independent experiments producing *n* = 20 fields of view (FOV) (control wells), n = 8 FOVs (“low” PMN concentration), *n* = 18 FOVs (“medium” PMN concentration), n = 14 FOVs (“high” PMN concentration). The data are unbalanced for the different neutrophil concentrations. **(B)** *S. aureus* cells were attached to the dish surface at an initial inoculum of ~10^2^ CFUs/cm^2^. To aggregate cells, *S. aureus* was incubated for 4 h after initial attachment (wells I and II). Single *S. aureus* cells were attached in control wells (wells III and IV) at the 4 h time point for comparison to non-aggregated cells. PMNs were then added to treatment wells (wells II and III) and imaged for 4 h. One well per condition (wells I and IV) was not treated with PMNs. *N* = 6 independent experiments with 12 FOVs per condition.

**Figure 2:**
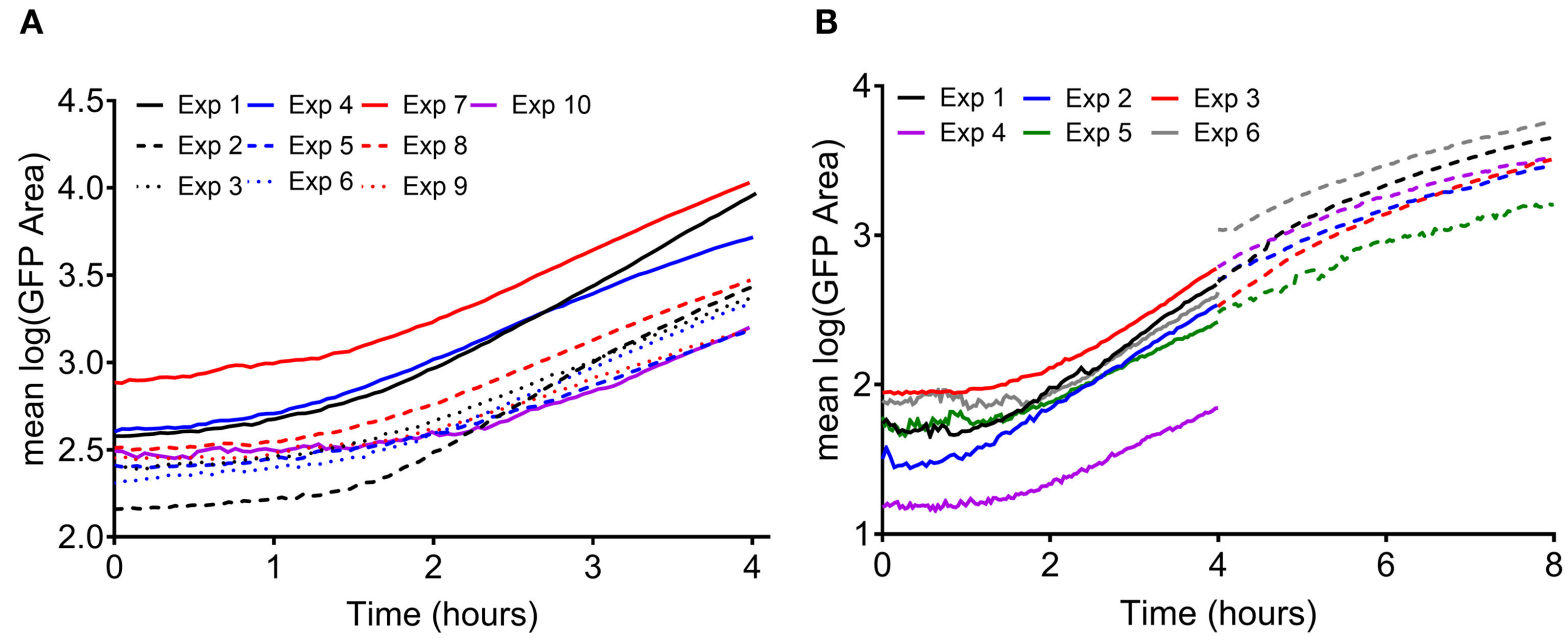
Measured growth of surface adherent *S. aureus* without an antimicrobial treatment. Each line represents the average of two FOVs from a given experiment. **(A)** Bacterial growth measured in density experiments (as in Figure 1A, well I). The initial inoculum was measured to be 1.83 ± 1.59 × 10^3^ CFUs/cm^2^. **(B)** Bacterial growth measured in head start experiments (as in Figure 1B). Solid lines indicate control wells in which single cells were attached and observed for 4 h (well IV in Figure 1B). Dashed lines indicate wells in which bacteria were given 4 h to grow prior to the start of imaging, then observed for 4 h (hours 4–8, well I in Figure 1B). The initial inoculum was measured to be 1.93 ± 0.70 × 10^2^ CFUs/cm^2^.

**Figure 3:**
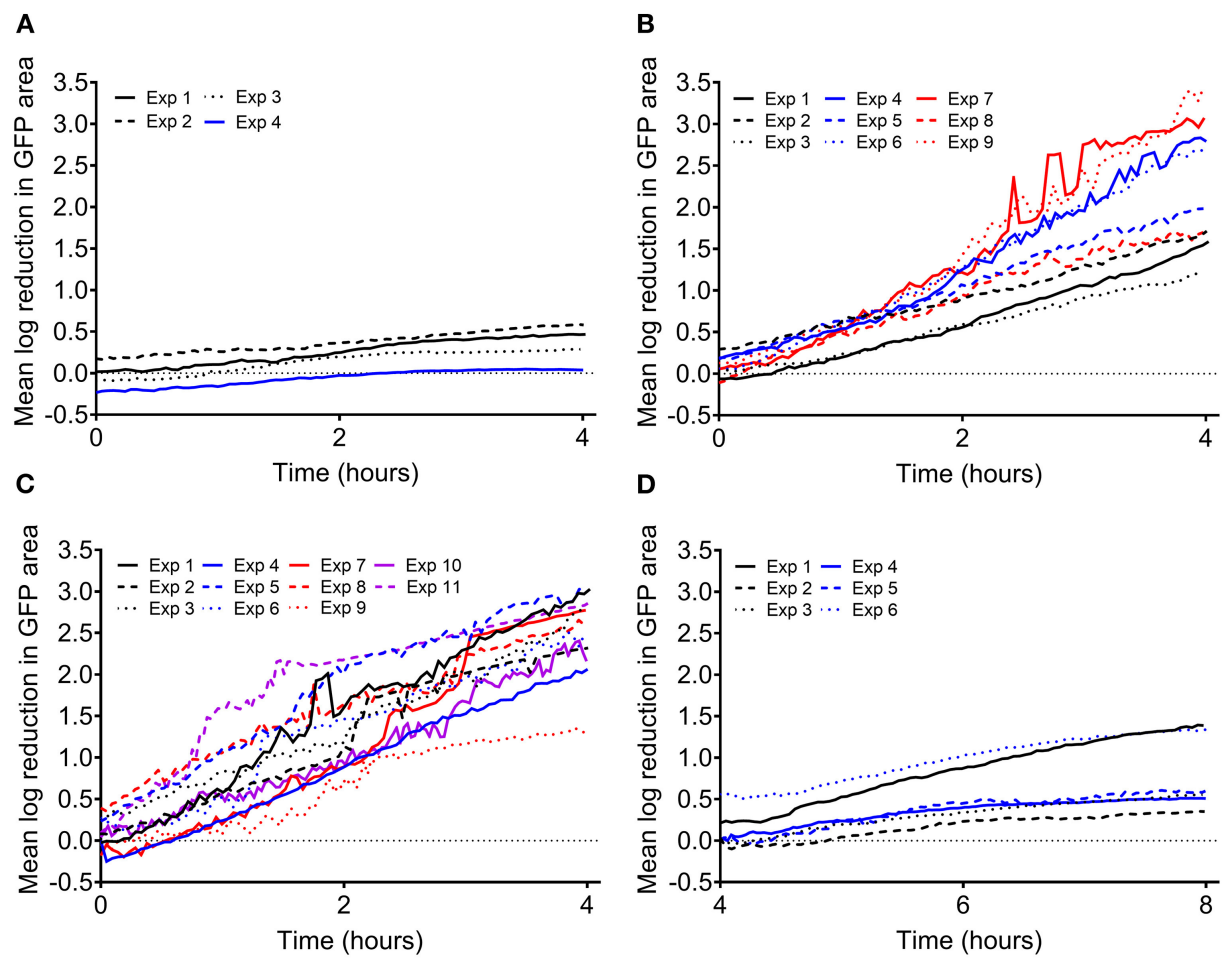
Log reduction in surface attached *S. aureus* biomass during PMN challenge compared to the average of the two corresponding control FOVs (no PMNs). Each line represents the average of two FOVs from a given experiment unless otherwise noted. Data shown from **(A)** density experiments with a low number of PMNs (2.39 ± 1.13 × 10^3^ PMNs/cm^2^, 8 FOVs, depicted in Figure 1A, well II) and **(B)** density experiments with a medium number of PMNs (1.53 ± 1.25 × 10^4^ PMNs/cm^2^, 18 FOVs, depicted in Figure 1A, well III). **(C)** Data shown from density experiments with high PMNs (Exps 1–5, 2.57 ± 1.18 × 10^4^ PMNs/cm^2^, 14 FOVs, depicted in Figure 1A, well IV) and control wells from head start experiments containing single cells and PMNs (Exps 6–11, 1.09 ± 0.31 × 10^4^ PMNs/cm^2^, 12 FOVs, depicted in Figure 1B, well III). Except for experiment 5, which contains 6 FOVs of the same condition, each line represents the average of two FOVs from a given experiment. **(D)** Data shown from head start experiments where aggregated bacteria were challenged with PMNs (1.72 ± 0.93 × 10^4^ PMNs/cm^2^, 12 FOVs, depicted in Figure 1B, well II).

Previous and ongoing studies in our lab have utilized a time lapse imaging system to analyze interactions between human PMNs and surface adherent *S. aureus* (Ghimire et al., 2019; Pettygrove et al., 2021). Data were mined from two types of experiments that were performed to investigate the effects of PMN density and *S. aureus* aggregate size on the overall clearance of *S. aureus* from a glass surface. We will refer to these two experiment setups as “density” and “head start” experiments, respectively (Figure 1). Extensive time-lapse microscopy data of bacterial growth spanning an 8-h time frame and several corresponding treatment conditions were collected for each of the described experiments (Figures 2, 3). The overarching results and interpretation of these experiments will not be discussed at length here but are the subject of a separate manuscript (Pettygrove et al., 2021). For density experiments it was observed that a high concentration of PMNs was required to clear adherent *S. aureus* with the “medium” and “high” treatment conditions resulting in significant clearance of bacteria (Figures 3A–C). Head start experiments demonstrated that aggregated *S. aureus* becomes highly resilient to PMN clearance and bacteria will persist through PMN challenge (Figure 3D). The relative contributions of inter-experimental, inter-FOV, and inter-timepoint variances at distinct phases in the bacterial growth cycle (namely during lag phase, early exponential phase, and late exponential phase) were all calculated from the collected time lapse microscopy data.

### Analysis of Untreated Biofilm

#### Variability of Early *S. aureus* Biofilm Colonization

Variability in observed GFP area, a surrogate for viable biomass (Schwartz et al., 2009), was analyzed over time for experiments at two inoculation concentrations for 4 h time periods (Figure 2). Bacterial growth was consistent between experiments, containing a lag phase, early exponential phase, and in the case of the “head start” experiments (Figure 2B), a late exponential phase. The repeatability standard deviation (SD) was calculated (data from Figure 2) for each hourly period from 0 to 8 h (hours 4–8 from experiments with a head start only), broken out by the inoculation level (Supplementary Table S1). Although the biomass area increases dramatically over time as the biofilm aggregates grow (Figure 2), the repeatability SDs and the proportions of variance were similar for the two inoculum levels within the lag or early exponential phase. The observed variance was also similar for each of the hourly periods within each phase, regardless of inoculation concentration (i.e., in Figure 2, the spread in the curves for the experiments is about the same over time). Thus, the data were pooled across inoculum levels and hours for each phase of growth. A single repeatability SD and components of variance of the log(area) were calculated for each phase of growth (Table 1). Overall, variation decreased as cells progressed through exponential phase. Thus, seeding and culture conditions may affect variation more during the early periods of biofilm colonization. It is crucial to have side-by-side controls in all experiments that are highly repeatable, otherwise the log reductions can be affected (Stewart, 2015). The level of variability of the untreated controls from our experiments is acceptably small (a repeatability SD of untreated controls <½ log is acceptable) compared to other methods in the peer reviewed literature that have been standardized with AOAC International (Tilt and Hamilton, 1999) or ASTM International (Parker et al., 2014; Goeres et al., 2019, 2020; Allkja et al., 2021).

#### Design Recommendations

After calculating the variation in *S. aureus* biomass during different phases of growth (Table 1), the improvement in the standard error of the mean log_10_(GFP Area) (SEM) was estimated under different experimental designs using the formula for SEM outlined in equation (1) in the methods. Given the SEM, we then predicted the margin of error (MOE) for a 95% confidence interval for the mean log(GFP Area) of untreated biofilms under varied numbers of replicate experiments (1–6), FOVs (1, 2, 6) and time rates of acquisition (10, 40 images per hour) (Table 2). Larger SEM and MOE values indicate more imprecision when estimating biomass from an image. Unsurprisingly, results for early exponential phase data indicate that at least three experiments should be performed to reach a MOE below 1 log(GFP area). While one FOV may be acceptable, exceeding two FOVs per experiment yields only mild returns with regards to increasing precision, suggesting two FOVs may be optimal. Additionally, increasing the temporal resolution only negligibly increases precision, so utilizing fewer timepoints per hour for temporal studies may be advantageous to reduce fluorophore photosensitivity or phototoxicity, especially when exciting with ultraviolet light. Assessments of different experimental designs for lag and late exponential phase growth data produced similar conclusions (Table 2). Overall, the improvement in precision diminishes with increased numbers of experiments or FOVs in each experiment. However, there is a much greater decrease in MOE when more experiments are performed, even when fewer FOVs are imaged in each experiment compared to fewer experiments with a large number of FOVs in each. For example, when estimating the mean log_10_(GFP area) of the untreated control biofilms during the early exponential phase at 95% confidence, consider a design utilizing two replicate experiments, two FOVs, and ten images per hour. Tripling the number of FOVs to six only yields a 9.3% reduction in the margin of error, while performing an additional experiment with two FOVs in each experiment results in a 62.3% reduction in the margin of error (Table 2). Conversely, performing six experiments with two FOVs and 10 images per hour reduces the margin of error by 81.5%, but requires a 3-fold increase in effort. Thus, our results suggest minimal benefit to utilizing a large number of FOVs with fewer experiments and instead recommend increasing the number of experiments performed when observing untreated biofilms during the early stages of colonization and development. However, the improvement in precision from performing more experiments must be weighed against the time and resource commitment required for those repetitions.

**Table 2:** Expected margin of error for a one-sided 95% confidence interval for the mean log_10_(GFP area) at different phases of growth and a variety of experimental parameters for untreated controls.

**Lag**	**FOV**	**1**	**1**	**2**	**2**	**6**	**6**
	**Frames**	**10**	**40**	**10**	**40**	**10**	**40**
Experiment number	1	20.0167	19.9466	16.2652	16.2221	13.1839	13.1661
	2	2.1765	2.1689	1.7686	1.7639	1.4335	1.4316
	3	0.8211	0.8182	0.6672	0.6654	0.5408	0.5401
	4	0.5723	0.5703	0.465	0.4638	0.3769	0.3764
	5	0.4639	0.4623	0.377	0.376	0.3056	0.3051
	6	0.4016	0.4002	0.3264	0.3255	0.2645	0.2642
**Early exponential**	**FOV**	**1**	**1**	**2**	**2**	**6**	**6**
	**Frames**	**10**	**40**	**10**	**40**	**10**	**40**
Experiment number	1	13.1355	13.0347	11.6722	11.6156	10.5849	10.5641
	2	1.4283	1.4173	1.2692	1.263	1.1509	1.1487
	3	0.5388	0.5347	0.4788	0.4765	0.4342	0.4333
	4	0.3755	0.3726	0.3337	0.3321	0.3026	0.302
	5	0.3044	0.3021	0.2705	0.2692	0.2453	0.2448
	6	0.2636	0.2615	0.2342	0.2331	0.2124	0.212
**Late exponential**	**FOV**	**1**	**1**	**2**	**2**	**6**	**6**
	**Frames**	**10**	**40**	**10**	**40**	**10**	**40**
Experiment number	1	8.9894	8.7664	7.6221	7.4911	6.554	6.5035
	2	0.9774	0.9532	0.8288	0.8145	0.7126	0.7071
	3	0.3687	0.3596	0.3126	0.3073	0.2688	0.2668
	4	0.257	0.2506	0.2179	0.2142	0.1874	0.1859
	5	0.2083	0.2032	0.1767	0.1736	0.1519	0.1507
	6	0.1804	0.1759	0.1529	0.1503	0.1315	0.1305

*The table shows that when quantifying untreated biofilms with a mean log_10_(GFP Area) during lag phase, at least 4 experiments with 2 FOVs each must be performed to get the error bars less than one half log. For early exponential phase, at least 3 experiments with 2 FOVs in each or 4 experiments must be performed while at least 3 experiments must be performed for late exponential phase*.

### Analysis of Treated Biofilm

#### Variability of PMN Clearance of Nascent *S. aureus* Biofilm

**Figure 4:**
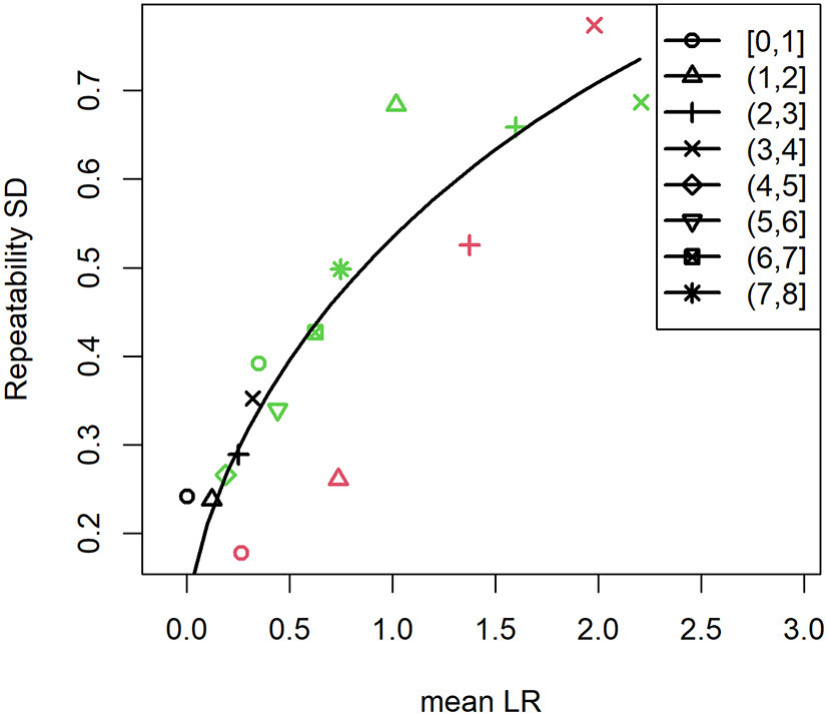
Repeatability standard deviation increases with the observed log reduction. Symbol shapes correspond to the observation time period (h) while colors indicate the concentration of PMNs that were used to challenge *S. aureus*. Black symbols indicate low PMN concentration (Figure 3A), red symbols indicate medium PMN concentration (Figure 3B), and green symbols indicate high PMN concentration (Figures 3C,D). Solid line represents the quadratic trend in the repeatability variance. Brackets indicate that the value is included in the given time range while parentheses indicate that the value is excluded.

By analyzing the results of experiments where surface attached *S. aureus* cells were challenged by human PMNs, we can make experimental design recommendations for studying the effect of an antimicrobial treatment on the viability of early biofilms. The two experimental setups (Figure 1) allowed us to analyze two different treatment scenarios. Experiments where the PMN density was varied (Figure 1A) can serve as an analog to changing the concentration of an antimicrobial treatment while the “head start” experiments (Figure 1B) can be modeled as application of a treatment at two different stages in biofilm development (in this case, lag and late exponential phase). It is important to note that discovery and subsequent killing of sparsely seeded *S. aureus* cells on a surface is a stochastic process that is highly dependent on the PMN concentration, unlike an antibiotic treatment that is comparatively homogenously applied (Ghimire et al., 2019). We calculated LRs in GFP area within each treatment FOV compared to the average of the two corresponding control FOVs at each time point. For PMN challenges against single cell *S. aureus*, as PMN density was increased, we observed higher LRs in GFP area, but increasing variability as the magnitude of the LR increased (Figures 3A–C). We similarly observed higher variability in LR in the efficacious “high PMN treatment” against single cells as compared to the relatively ineffective “high treatment” against aggregated cells (Figure 3D). Repeatability SD did not change markedly over time, but did increase with the observed LR, regardless of the experimental setup (Density vs. Head Start) (Figure 4). Intuitively, a treatment that is poorly efficacious (Figures 3A,D) will consistently result in small reductions and be more repeatable (Parker et al., 2018a). On the other hand, for a treatment that is highly efficacious and consistently kills all attached cells, we would expect a high level of repeatability as well (such a highly efficacious treatment was not tested in our experiments). Here, we observe that treatments of middling efficacy are highly variable. Thus, experiments where a treatment is expected to be highly efficacious or very ineffective will require fewer repetitions while moderately effective treatments require more. Our statistical model for the LRs explicitly accounts for differences among experiments. Because multiple donors contributed PMNs, the repeatability variance in Figure 4 may be larger than what would be expected if all PMNs were collected from the same donor. Nonetheless, the repeatability SDs for LRs that were observed in our system were <1 log and hence acceptable according to ASTM International and AOAC international (Parker et al., 2018a).

#### Design Recommendations

In our experiments measuring killing of attached *S. aureus* cells by human PMNs, we observed that the variance changes as a function of the LR regardless of inoculation level and time (Figure 4 and Supplementary Table S2). As with the data from control wells, we used these results to evaluate how frequently to image, how many experiments to conduct, and how many FOVs to utilize per experiment in order to generate estimates of the mean LR with statistical confidence. The first step was to calculate the SEM for the mean LR using equation (1) from the estimated variance components for experiment, FOV and time (Supplementary Figure S1). Using these values, the expected MOE was assessed for experimental designs for treatments that have a mean LR = 0.5, 1, or 2 over a wide range of experiment numbers, FOV numbers, and temporal resolutions (Table 3). Because the variance increases as the mean LR increases (Figure 4), the SEM increases as the LR increases. Nevertheless, this does not translate into more experimental effort to precisely estimate the mean LR for moderately efficacious treatments (with mean LR = 2) compared to less efficacious treatments (with mean LRs ≤ 1) because the magnitude of the LR is less for less efficacious treatments. For an expected LR = 0.5, at least 4 experiments are required to detect a significant kill, while for LR = 1 or 2, only 3 experiments are necessary (Table 3). While the minimum number of experiments required to detect a significant kill does not change based on the number of FOVs (except when using 6 FOVs when LR = 0.5), modest improvements in the MOE are expected when at least 2 FOVs are utilized and may provide considerable benefit to the researcher. Providing an expected LR is a standard input for sample size calculations. The only difference in our approach from the standard approach is that in addition to using the expected LR as the margin of error input into the sample size calculator, we also use the expected LR to look up the variability of the LR (*via* Figure 4). Using the standard sample size calculator approach, the variability would be assumed to be the same for all LR values, which clearly is not the case for these efficacy data (see Figure 4 and the review in Parker et al., 2018a).

**Table 3:** Expected margin of error (MOE) for a 1-sided 95% confidence interval for the mean log reduction in GFP area at a variety of experimental parameters and when LR = 0.5 (i.e., when percent reduction is 68%), when LR = 1 (i.e., when percent reduction is 90%), and when LR = 2 (i.e., when percent reduction is 99%).

**LR = 0.5**	**FOV**	**1**	**1**	**2**	**2**	**6**	**6**
	**Frames**	**10**	**40**	**10**	**40**	**10**	**40**
Experiment number	1	16.2871	16.2155	13.872	13.83	11.9948	11.9786
	2	1.7681	1.7604	1.506	1.5014	1.3022	1.3004
	3	0.6681	0.6651	0.569	0.5673	0.492	0.4913
	4	0.4656	0.4636	0.3966	0.3954	0.3429	0.3425
	5	0.3775	0.3758	0.3215	0.3205	0.278	0.2776
	6	0.3268	0.3254	0.2783	0.2775	0.2407	0.2403
**LR = 1**	**FOV**	**1**	**1**	**2**	**2**	**6**	**6**
	**Frames**	**10**	**40**	**10**	**40**	**10**	**40**
Experiment number	1	22.1521	22.1521	18.9751	18.9751	16.5211	16.5211
	2	2.4048	2.4048	2.06	2.06	1.7935	1.7935
	3	0.9086	0.9086	0.7783	0.7783	0.6777	0.6777
	4	0.6333	0.6333	0.5425	0.5425	0.4723	0.4723
	5	0.5134	0.5134	0.4398	0.4398	0.3829	0.3829
	6	0.4445	0.4445	0.3807	0.3807	0.3315	0.3315
**LR = 2**	**FOV**	**1**	**1**	**2**	**2**	**6**	**6**
	**Frames**	**10**	**40**	**10**	**40**	**10**	**40**
Experiment number	1	29.3802	28.4735	24.0585	23.5071	19.7287	19.5059
	2	3.1895	3.0911	2.6118	2.5519	2.1418	2.1176
	3	1.2051	1.1679	0.9868	0.9642	0.8092	0.8001
	4	0.8399	0.814	0.6878	0.672	0.564	0.5577
	5	0.6809	0.6599	0.5576	0.5448	0.4572	0.4521
	6	0.5895	0.5713	0.4827	0.4717	0.3959	0.3914

*The MOE was calculated from equation (1) using the variance components from Supplementary Figure 1 for each LR value. The table shows that when studying a treatment with a mean LR = 0.5, at least 3 experiments, each with 6 fields of view; or 4 experiments with any number of fields of view must be performed to be able to detect a statistically significant kill. When studying a treatment with a mean LR = 1 or LR = 2, at least 3 experiments must be performed to be able to detect a statistically significant kill. Because the variance due to time is 0 for LR = 1 (Supplementary Figure 1), there is no effect on the margin of error due to differences in the number of images acquired*.

#### Validation of Design Recommendations

To determine how well Table 3 predicts the margin of error, we assessed a second dataset utilizing a 10 μg/mL gentamicin treatment rather than PMN treatment. Similar to the previously described experiments, *S. aureus* was grown for 3 h, then treated with gentamicin for 4 h and imaged via confocal microscopy. We expected a LR of around 1 after 4 h of contact time and a repeatability SD of around 0.5 (Figure 4). Table 3 suggests that 3 independent experiments with 1 FOV each would be sufficient to detect a significant kill. The dataset that we analyzed contained 3 independent experiments with 3 control FOVs and 3 treated FOVs in each experiment, allowing us to assess the MOE for many possible combinations of 3 experiments with 1 control FOV and 1 treated FOV (27 possible control FOV combinations and 27 possible treatment FOV combinations yielding 729 possible LRs). We simulated 1,000 studies with this design via bootstrap sampling and calculated the mean LR, MOE, and lower 95% confidence limit for each experimental design (Supplementary Figure S2). Overall, the gentamicin treated data was less variable (mean LR = 0.80, repeatability SD = 0.12) than the neutrophil treated data (repeatability close to 0.5 according to Figure 4), resulting in MOEs much smaller than the predicted value of 0.91 (Table 3). In 100% of the simulations, the design produced a statistically significant kill (Supplementary Figure S2C). This indicates that the recommendation of 3 experiments and 1 FOV, while conservative, was sufficient for detecting the mean LR with 95% confidence using this experimental setup (gentamicin treatment rather than PMN treatment).

## Discussion

Real-time CLSM non-invasively provides quantitative insight into the dynamics of living biofilms (Stewart et al., 2009; Davison et al., 2010; McLoon et al., 2011; Hung et al., 2013; Franklin et al., 2015; Ghimire et al., 2019; Pettygrove et al., 2021). Continued exploration in this area by our lab has yielded an abundance of time-lapse microscopy data that can inform the proper design of microscopy studies (Ghimire et al., 2019; Pettygrove et al., 2021). The data included are particularly suitable for assisting with the experimental design of studies investigating initial bacterial attachment, early biofilm formation, and biocidal treatments of bacteria in this stage. However, the development of criteria for selecting the appropriate number of FOV that are required to produce representative quantitative results have been overlooked for this type of data collection. In this paper, we aimed to retrospectively utilize time-lapse data to provide quantitative guidance on the aspects of experimental design such as the numbers of experiments, fields of view, and images over time to provide an acceptable level of precision when estimating biomass on a surface.

In the presented data, variance for untreated controls representative of early biofilm formation (i.e., early colonization), depended on the growth phase of interest (lag or exponential in our data). The repeatability SD tended to decrease as cells entered exponential phase (Table 1). Thus, when designing an imaging experiment to study biofilm colonization, the user should primarily consider which phase of growth will be monitored and adapt the number of experiments, FOVs, and time points to include in the experimental design accordingly. The increased repeatability SD observed during the lag phase compared to early and late exponential is likely in part due to the high amounts of heterogeneity in the initial observed growth. *S. aureus* cells were seeded onto the surface in PBS, rather than a nutrient rich media, resulting in differential recovery from lag phase for individual colonies, leading to high intra-FOV variation during lag phase as attached cells began to readjust to the media conditions. Thus, initial seeding conditions may affect variability as well and must be assessed for each system. For experiments where the measured output is a reduction in viable bacteria (GFP area in these experiments), the expected LR is required to estimate the appropriate number of experiments, FOVs, and time points. It is to be expected that the variability will be markedly different for different laboratory systems (Parker et al., 2018a; Stewart and Parker, 2019). Nonetheless, here we present a methodology for others to follow to estimate the variance in their systems and allow experimental design assessments.

As it pertains to repeatability, experimental bias is a significant obstacle for imaging studies. Sources of bias in imaging experiments can include the selection of representative regions of interest, image collection parameters, and the number of visualized FOVs (Lee and Kitaoka, 2018). Relatively arbitrary recommendations have been made for approaches to minimize bias such as visualizing a greater number of FOV, increasing the size of the visualized area, and imaging multi-well plates (Patel and McGhee, 2013; Lee and Kitaoka, 2018). There is limited suitability of some of these recommendations as it pertains to real-time biofilm imaging due to phototoxicity and the need for rapid image acquisition. As is common, the fields of view that we analyze here were not randomly chosen since the bacterial attachment was not homogenous across the surface. Statistical predictions and confidence intervals from hypothetical experimental designs, as we do here, presume that the data collected are representative of some population of all possible fields of view that could have been selected. Usually, representative data are assured by random sampling, in this case randomly selecting fields of view, wherein the population of interest is all possible fields of view. True random sampling is not typically performed in imaging studies as many fields of view are empty, especially during attachment or early biofilm formation. For the data collected here, the population of interest was all fields of view that contained some population of PMNs and biofilm aggregates, as determined by the microscopist. Fields of view were selected to maximize the number of PMN-*S. aureus* interactions that could be imaged, thus most fields of view were selected because of a high number of *S. aureus* aggregates present. While FOV selection is subjective and often dependent on the microscopist, our results are a starting point for estimating the sources contributing to the variance in observed biomass and designing microscopy experiments. There are methods, however, to reduce bias due to the subjective selection of fields of view. For example, in the ecological literature, there are adaptive sampling techniques that begin by randomly selecting locations for observation, i.e., randomly selecting a FOV (Thompson, 1990). When the ecologist (or microscopist) moves and encounters a new location (or FOV) of interest, the resulting statistics can be “adapted” to account for this deviation in sampling. Implementation of adaptive sampling that adjusts the image statistics is a matter for future research. Our lack of random sampling is a potential weakness of our work. We recommend adaptive sampling of FOVs to minimize bias due to subjective FOV selection, but until the tools for CLSM have been developed, the next best approach to alleviate bias is to randomly select fields of view from the surface being imaged.

Our results, not surprisingly, point to the need to perform at least three experiments in most scenarios. For a given growth phase or expected LR, a substantial contribution to variance in biomass is due to differences between experiments (Table 1 and Supplementary Figure S1). Furthermore, our results indicate that a single FOV in each experiment may be sufficient, as the resulting reduction in variance from increasing FOVs is small. Most experimenters will want to collect at least two FOVs, but our results suggest that there is only minimal benefit to using more than two in most cases. Furthermore, as neutrophil challenge is a relatively heterogeneous antimicrobial treatment as compared to antibiotic therapy, it is plausible that the inter-FOV variance in our system is higher than might be observed in an antibiotic treated system. Lastly, our results demonstrate that there is not substantial increase to precision by increasing the temporal resolution of the acquired images in the video. In our variance predictions, little difference was seen between conditions utilizing 10 images per hour compared to 40 images per hour, suggesting an experimenter should utilize the temporal resolution that is best for their sample as the effect on their data quality will be minimal, barring phototoxicity. For visualizing rapid processes, high temporal resolution has an obvious benefit, however lower temporal resolution can both decrease phototoxicity and downstream image analysis burden. We observed little phototoxicity or fluorophore bleaching in our system despite a frequent image capture rate, however each experimenter should decide how rapidly to image based on the organism, fluorophores, and conditions necessary for their work. It is to be expected that a study of a different organism, even when using the same method, will produce results that have different levels and sources of variation than *S. aureus*. For example, a motile organism may enter and leave a FOV, while other organisms may have less predictable attachment mechanics. Similarly, different growth or treatment conditions would introduce different levels and sources of variability. These factors could lead to different inter-experimental, inter-FOV, or inter-timepoint variance compared to the values measured in our study. Therefore, we strongly recommend that researchers generate their own pilot data using their own methods and organisms and use the resulting variance component estimates to complete equation (1) via the included Excel tool to generate study designs. However, in the absence of available pilot data, which unfortunately is many times the case, the study designs (in Tables 2, 3) calculated from our data can serve as a starting point, with the understanding that there are likely differences between the system we present here and systems used by other researchers. Regardless of the data used, the analysis framework presented in this manuscript can help other researchers design statistically sound experiments based on a quantitative understanding of the contributors to variance in their system. Put another way, while the laboratory methodology may vary, the same statistical approach can be utilized to assess study designs.

The Supplementary Materials contains an Excel tool that allows the user to predict the number of experiments, FOVs, and images per hour (i.e., an experimental design) to estimate experimental parameters calculated from images of early biofilms at 95% confidence. The two parameters we focus on are the log transformed surface coverage [i.e., log_10_(GFP area)] of untreated control biofilms or the LR measure of treatment efficacy. The tool requires estimates of the variance components due to experiments, FOVs and time for different experimental setups. For untreated control biofilms, the variance estimation will depend on the growth phase of interest. For treated biofilms, the variance estimation will depend on the expected LR.

As the field of quantitative imaging progresses there is a growing need for increased reporting on imaging acquisition parameters, processing, figure generation, and statistical analysis. The results reported here provide a statistical approach for deciding on appropriate replication to perform in imaging-based experiments during early biofilm colonization. Results from the presented approach also provide an efficient way for communicating methodology and statistical rigor from imaging experiments to the scientific community. While the variance observed will depend on one's specific experimental setup, these results do demonstrate the value of prioritizing experimental repetitions over high numbers of FOVs. The results (Tables 2, 3) also demonstrate the diminishing returns gained from increasing experimental repetitions and FOVs, which can help researchers decide how best to balance workload and the expected quality of data. The data mined for these analyses is derived from experiments examining sparsely seeded surfaces, thus future experiments should examine densely seeded surfaces and other measurable outputs such as volume rather than just area or surface coverage. In addition to early colonization studies there is a large component of biofilm research that is conducted on mature biofilms, hence future work should investigate the variability observed in imaging mature biofilms. Nonetheless, the statistical approach presented herein could be applied to biofilms at different life stages or different organisms and assays. We intend for this method to serve as a framework to be utilized with any pilot data examining biofilm growth to better inform the experimental design. Together, the results described in this paper can act as a roadmap to assist researchers in the design of microscopy-based studies of biofilm colonization to ensure that acquired data is representative of the sample and statistically robust.

## Data Availability Statement

The raw data supporting the conclusions of this article will be made available by the authors, without undue reservation.

## Ethics Statement

The studies involving human participants were reviewed and approved by Montana State University Institutional Review Board. The patients/participants provided their written informed consent to participate in this study.

## Author Contributions

BP designed and conducted experiments, performed data analysis, and drafted the manuscript. HS conceived the study, performed data analysis, drafted the manuscript, and supervised the study. KP performed blood draws and PMN isolations and revised the manuscript. JV and PS designed experiments, provided laboratory resources, revised the manuscript, supervised the study, and acquired funding. AP conceived the study, performed the statistical analysis, drafted the manuscript, and supervised the study. All authors contributed to the article and approved the submitted version.

## Funding

This work was supported by grants U54GM115371, RO1AI149491, and 1R56AI155692 from the National Institutes of Health. Imaging was made possible by microscope facilities at the Center for Biofilm Engineering, which were supported by funding from the National Science Foundation MRI Program and the M. J. Murdock Charitable Trust.

## Conflict of Interest

The authors declare that the research was conducted in the absence of any commercial or financial relationships that could be construed as a potential conflict of interest.

## Publisher's Note

All claims expressed in this article are solely those of the authors and do not necessarily represent those of their affiliated organizations, or those of the publisher, the editors and the reviewers. Any product that may be evaluated in this article, or claim that may be made by its manufacturer, is not guaranteed or endorsed by the publisher.
