# Supplementary material for: Experimental Designs to Study the Aggregation and Colonization of Biofilms by Video Microscopy With Statistical Confidence
Source: Front Microbiol. 2022 Jan 13;12:785182. doi: 10.3389/fmicb.2021.785182 (PMC8793059; doi:10.3389/fmicb.2021.785182)
Supplement: Supplementary file 1 [file Table_1.DOCX]

**Supplementary Material**

**Table S1.** Mean and repeatability SD of the log_10_(area), by hourly periods and inoculum levels, of the control data in Figure 2. The yint and rate parametrize the line log_10_(Area) = yint + rate×Time in each hourly period. The mean time and mean log_10_(Area) in each time period are also given in separate columns. The last columns provide, for each hourly period, the proportion of variance attributable to each of experiment, field of view, and frames as well as the repeatability SD. EE indicates early exponential phase and LE indicates late exponential phase.

| Phase | Log Inoc. | Period | yint | rate | Mean Time | Mean Log Area | Prop. Exp | Prop. FOV | Prop. Time | SD. Repeat |
| --- | --- | --- | --- | --- | --- | --- | --- | --- | --- | --- |
| Lag | 2 | [0,1] | 1.651 | 0.026 | 0.497 | 1.664 | 0.766 | 0.216 | 0.018 | 0.304 |
| Lag | 2 | (1,2] | 1.495 | 0.164 | 1.509 | 1.743 | 0.586 | 0.396 | 0.019 | 0.298 |
| EE | 2 | (2,3] | 1.235 | 0.304 | 2.503 | 1.995 | 0.467 | 0.530 | 0.004 | 0.323 |
| EE | 2 | (3,4] | 1.233 | 0.310 | 3.506 | 2.322 | 0.567 | 0.430 | 0.003 | 0.353 |
| LE | 2 | (4,5] | 1.559 | 0.288 | 4.497 | 2.852 | 0.351 | 0.628 | 0.021 | 0.230 |
| LE | 2 | (5,6] | 1.852 | 0.230 | 5.509 | 3.119 | 0.464 | 0.527 | 0.009 | 0.213 |
| LE | 2 | (6,7] | 2.287 | 0.157 | 6.503 | 3.308 | 0.517 | 0.477 | 0.005 | 0.209 |
| LE | 2 | (7,8] | 2.462 | 0.133 | 7.506 | 3.600 | 0.559 | 0.438 | 0.003 | 0.208 |
| Lag | 3 | [0,1] | 2.472 | 0.063 | 0.492 | 2.503 | 0.484 | 0.511 | 0.005 | 0.233 |
| Lag | 3 | (1,2] | 2.316 | 0.207 | 1.506 | 2.628 | 0.541 | 0.449 | 0.010 | 0.258 |
| EE | 3 | (2,3] | 1.985 | 0.378 | 2.504 | 2.931 | 0.621 | 0.372 | 0.007 | 0.289 |
| EE | 3 | (3,4] | 1.992 | 0.377 | 3.510 | 3.314 | 0.662 | 0.334 | 0.004 | 0.324 |

**Table S2.** Mean and repeatability SD of the log reductions (LR) in Figures 3, by hourly period. The results in this table are shown graphically in Figure 4. The yint and rate parametrize the line LR = yint + rate×Time in each hourly period. The last columns provide, for each hourly period, the proportion of variance attributable to each of experiment, field of view, and frames as well as the repeatability SD and the variance inflation factor. “Level” refers to the approximate concentration of PMNs in the FOV. EE indicates early exponential phase and LE indicates late exponential phase.

| Phase | Level | Log Inoc. | Period | yint | rate | Mean Time | Mean LR | Prop. Exp | Prop. FOV | Prop. Time | SD. Repeat | Inf. Fact |
| --- | --- | --- | --- | --- | --- | --- | --- | --- | --- | --- | --- | --- |
| Lag | Low | 3 | [0,1] | -0.040 | 0.092 | 0.483 | 0.005 | 0.054 | 0.937 | 0.009 | 0.242 | 5.181 |
| Lag | Low | 3 | (1,2] | -0.074 | 0.131 | 1.490 | 0.122 | 0.000 | 0.988 | 0.012 | 0.238 | 7.783 |
| EE | Low | 3 | (2,3] | -0.004 | 0.102 | 2.492 | 0.251 | 0.000 | 0.990 | 0.010 | 0.289 | 11.44 |
| EE | Low | 3 | (3,4] | 0.128 | 0.055 | 3.511 | 0.322 | 0.000 | 0.997 | 0.003 | 0.352 | 8.832 |
| Lag | Med | 3 | [0,1] | 0.056 | 0.425 | 0.491 | 0.265 | 0.208 | 0.668 | 0.123 | 0.178 | 6.197 |
| Lag | Med | 3 | (1,2] | -0.062 | 0.531 | 1.505 | 0.737 | 0.406 | 0.504 | 0.090 | 0.261 | 4.288 |
| EE | Med | 3 | (2,3] | -0.252 | 0.650 | 2.504 | 1.374 | 0.556 | 0.327 | 0.116 | 0.526 | 2.945 |
| EE | Med | 3 | (3,4] | 0.061 | 0.547 | 3.510 | 1.981 | 0.587 | 0.384 | 0.029 | 0.774 | 5.161 |
| Lag | High | 2 | [0,1] | 0.035 | 0.634 | 0.497 | 0.350 | 0.590 | 0.172 | 0.239 | 0.392 | 7.437 |
| Lag | High | 2 | (1,2] | 0.021 | 0.660 | 1.509 | 1.012 | 0.598 | 0.305 | 0.097 | 0.683 | 7.698 |
| EE | High | 2 | (2,3] | 0.202 | 0.559 | 2.503 | 1.600 | 0.462 | 0.909 | 0.045 | 0.658 | 3.977 |
| EE | High | 2 | (3,4] | 0.826 | 0.394 | 3.506 | 2.208 | 0.185 | 0.784 | 0.217 | 0.687 | 4.107 |
| LE | High | 2 | (4,5] | -0.778 | 0.215 | 4.497 | 0.190 | 0.505 | 0.466 | 0.029 | 0.266 | 8.132 |
| LE | High | 2 | (5,6] | -0.926 | 0.248 | 5.509 | 0.443 | 0.426 | 0.566 | 0.008 | 0.340 | 7.880 |
| LE | High | 2 | (6,7] | -0.153 | 0.120 | 6.503 | 0.626 | 0.472 | 0.521 | 0.007 | 0.427 | 12.05 |
| LE | High | 2 | (7,8] | 0.025 | 0.096 | 7.506 | 0.748 | 0.506 | 0.492 | 0.002 | 0.498 | 5.750 |

**
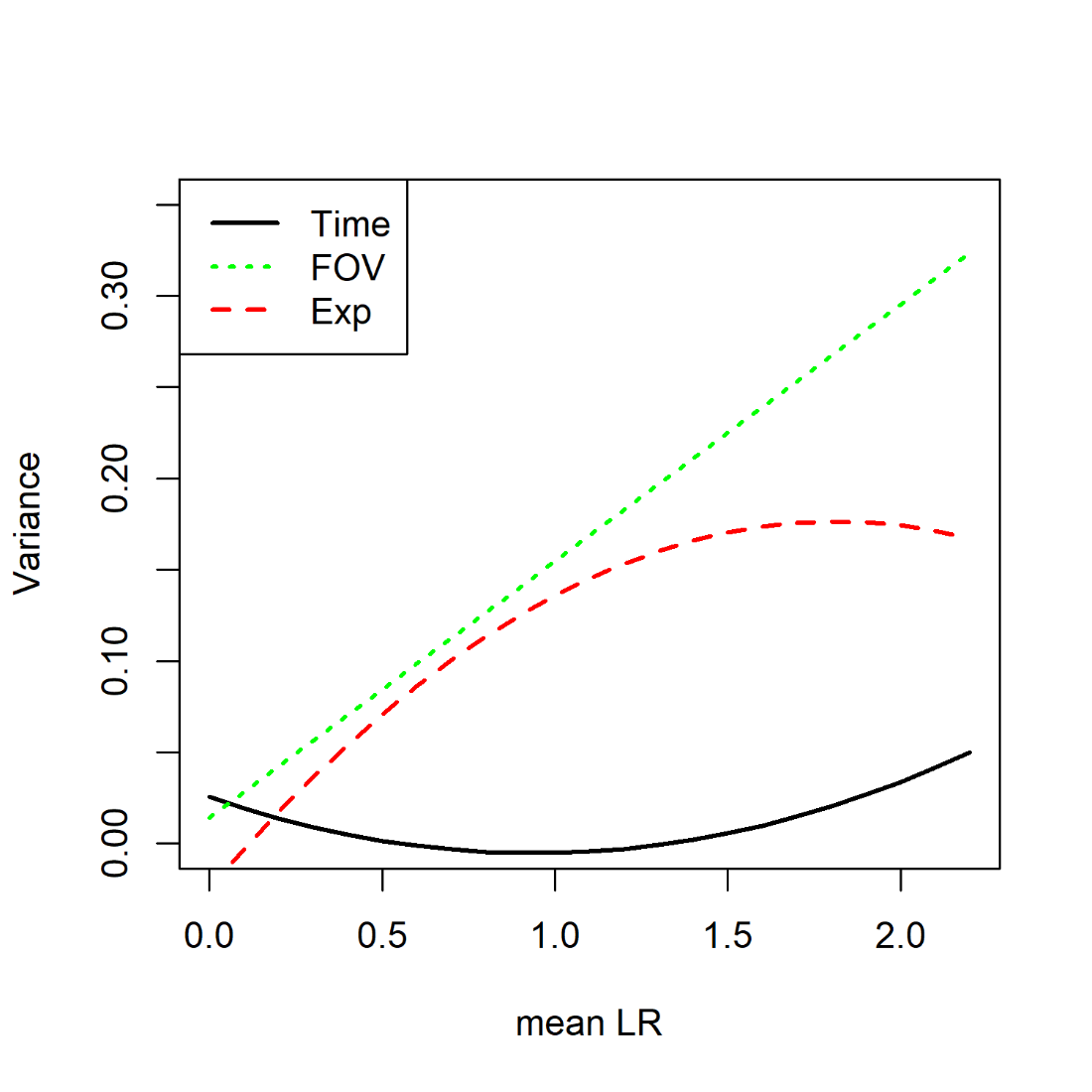
**

**Figure S1.** All components of variance increase with the observed log reduction. For the 3 LR values that we focus on for experimental designs, the variance due to experiment (Var_exp_), FOV (Var_FOV_) and time (Var_time_) are: 0.0708, 0.0844 and 0.0015 for LR = 0.5; 0.1358, 0.1547 and 0.000 for LR = 1; and 0.1743, 0.2953 and 0.0338 for LR = 2.

**
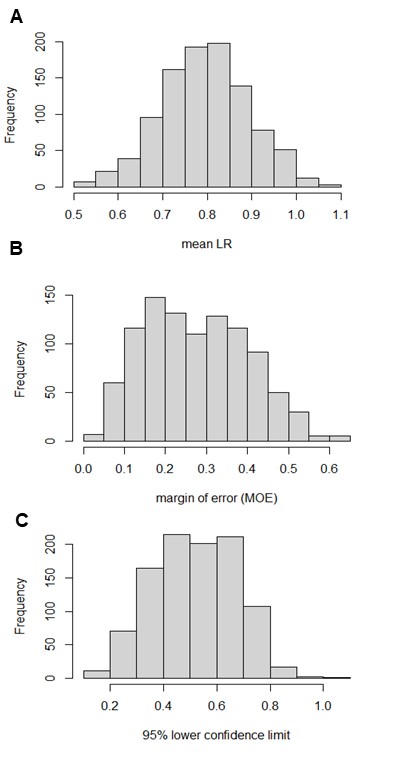
**

**Figure S2.** Assessment of recommended experimental design success by bootstrapping a secondary dataset. A hypothetical experimental design with expected LR = 1 was assessed based on the recommendation in Table 3 (3 experiments with 1 FOV each). A dataset (gentamicin treatment of *S. aureus*) containing 3 experiments with 3 FOVs each was bootstrapped 1000 times to simulate 1000 data sets with 3 experiments and 1 FOV each. (*A*) Histogram of the mean log reduction [LR = log_10_(*Control*) – log_10_(*Treated*)] for the simulated data sets. (*B*) Histogram of the margin of error (MOE) for the simulated data sets. (*C*) Histogram of the 95% lower confidence limit (LR – MOE) for the simulated data sets. Since 100% of the confidence limits were greater than 0, the 3-experiment and 1-FOV design was successful at estimating the mean LR with 95% confidence.
